# Zinc Deprivation as a Promising Approach for Combating Methicillin-Resistant *Staphylococcus aureus*: A Pilot Study

**DOI:** 10.3390/pathogens10101228

**Published:** 2021-09-23

**Authors:** Yomna A. Elhakim, Amal E. Ali, Alaa El-Dien M. S. Hosny, Nourtan F. Abdeltawab

**Affiliations:** 1 Department of Microbiology and Immunology, Faculty of Pharmacy, Cairo University, Cairo 11562, Egypt; yomna.elsayed@pharma.cu.edu.eg (Y.A.E.); doctor.alaa@hotmail.com (A.E.-D.M.S.H.); 2 Department of Microbiology and Immunology, Faculty of Pharmacy, Future University in Egypt, New Cairo 12311, Egypt; amal.emad@fue.edu.eg; 3 Department of Microbiology and Immunology, Faculty of Pharmacy, Modern University for Technology and Information (MTI), Cairo 12055, Egypt

**Keywords:** methicillin-resistant *S. aureus* (MRSA), zinc, biofilm, hemolysis, alternative antimicrobials, infection

## Abstract

Methicillin-resistant *Staphylococcus aureus* (MRSA) infections are a global health burden with an urgent need for antimicrobial agents. Studies have shown that host immune responses limit essential metals such as zinc during infection, leading to the limitation of bacterial virulence. Thus, the deprivation of zinc as an important co-factor for the activity of many *S. aureus* enzymes can be a potential antimicrobial approach. However, the effect of zinc deprivation on *S. aureus* and MRSA is not fully understood. Therefore, the current study aimed to dissect the effects of zinc deprivation on *S. aureus* hemolytic activity and biofilm formation through employing biochemical and genetic approaches to study the effect of zinc deprivation on *S. aureus* growth and virulence. Chemically defined media (CDM) with and without ZnCl_2_, was used to assess the effect of zinc deprivation on growth, biofilm formation, and hemolytic activity in methicillin-susceptible *S. aureus* (MSSA) RN6390 and MRSA N315 strains. Zinc deprivation decreased the growth of RN6390 and N315 *S. aureus* strains significantly by 1.5–2 folds, respectively compared to the zinc physiological range encountered by the bacteria in the human body (7–20 µM) (*p* < 0.05). Zinc deprivation significantly reduced biofilm formation by 1.5 folds compared to physiological levels (*p* < 0.05). Moreover, the hemolytic activity of RN6390 and N315 *S. aureus* strains was significantly decreased by 20 and 30 percent, respectively compared to physiological zinc levels (*p* < 0.05). Expression of biofilm-associated transcripts levels at late stage of biofilm formation (20 h) murein hydrolase activator A (*cidA*) and *cidB* were downregulated by 3 and 5 folds, respectively (*p* < 0.05) suggested an effect on extracellular DNA production. Expression of hemolysins-associated genes (*hld, hlb, hla*) was downregulated by 3, 5, and 10 folds, respectively, in absence of zinc (*p* < 0.001). Collectively the current study showed that zinc deprivation in vitro affected growth, biofilm formation, and hemolytic activity of *S. aureus*. Our in vitro findings suggested that zinc deprivation can be a potential supportive anti-biofilm formation and antihemolytic approach to contain MRSA topical infections.

## 1. Introduction

*Staphylococcus aureus* is implicated in community-acquired and nosocomial infections posing a health care burden [1,2,3]. Since the discovery of penicillin, *S. aureus* was one of the pathogens that developed resistance against most newly introduced antibiotics. *Staphylococcus aureus* was remarkably able to resist a number of antibiotics leading to the development of methicillin-resistant *S. aureus* (MRSA) and vancomycin-resistant *S. aureus* (VRSA) [4]. The emergence of resistant strains and their high prevalence worldwide has resulted in the difficult eradication of staphylococcal infections in health care facilities [5,6,7]. Enzymes like proteases, lipases, and nucleases enable *S. aureus* to obtain nutrients from host tissues to help bacterial growth [8]. Besides enzymes, cytotoxins including the four members of the hemolysins family help *S. aureus* evade the host immune system [9]. Not only does *S. aureus* produce toxins, but also it can persist in the human body using its ability of biofilm formation [10]. Cells within biofilm have a higher survival chance due to resistance to sweeping by frictional forces and difficulty of phagocytosis of cells within the biofilm. Moreover, the limitation of antibiotic diffusion into the biofilm community hinders the action of antibiotics [11,12,13].

A promising approach to combat staphylococcal infections is by mimicking the human immune responses that disrupt the ability of bacteria to grow. This includes mechanisms of limiting essential nutrients, a process known as nutritional immunity [14]. For instance, neutrophils can restrict the growth of *S. aureus* by creating an environment devoid of metals [15]. Utilizing metal ions as cofactors in basic metabolic pathways is essential for the survival of all living organisms whether it is the pathogenic organism or its host [16,17]. During evolution, the common need for these important nutrients directed the host–pathogen relationship into a competition over metal ions. To overcome pathogenic microbes, the host has developed several mechanisms to limit the levels of free metals. Meanwhile, pathogens have developed metal ions acquisition and transport mechanisms to overcome such adverse environments [18,19,20].

Impact of iron limitation on bacterial growth and how bacterial pathogens developed mechanisms to overcome such limitation has been studied [21,22,23]. The feasibility of limiting iron in the supportive treatment and prevention of bacterial infections has been explored. For instance, lactoferrin, an iron-binding glycoprotein, is used in the supportive treatment of *Helicobacter pylori* infections [24]. Zinc starvation is imposed inside the human immune cells by zinc-binding proteins, e.g., calprotectin and metallothionein, limiting infection [25,26,27]. However, a few studies have focused on dissecting the effects of zinc limitation on bacterial growth and virulence. One study showed that in liver abscesses immune cells deplete zinc and manganese to limit bacterial growth [15]. Another study showed that limiting zinc has an important role in controlling *H. pylori* infection thus modifying bacterial host interaction [28]. Velasco et al. (2018) suggested a possible impact of zinc on hemolysins activity in *Escherichia coli*, where zinc deprivation and zinc uptake regulator (zur) were involved in the expression of an alpha-hemolysin virulence factor in clinical isolates of uropathogenic *E. coli* [29]. However, the effect of zinc on host-pathogen interaction especially in gram-positive microorganisms in specific *S. aureus* remains not well-studied. 

Thus, the main goal of this pilot study was to identify the effect of zinc deprivation on the growth and virulence of *S. aureus*. *Staphylococcus aureus* growth, biofilm formation, and hemolysis activity were compared under the zinc physiological range encountered by the bacteria in the human body (7 and 20 µM) and in absence of zinc [30,31,32,33,34]. Zinc concentrations were chosen based on previous studies showing physiologically encountered levels in the body [35,36,37,38,39] and those inside immune cells where calprotectin and other proteins subject bacteria to complete deprivation of zinc [15,25]. Both biochemical and genetic approaches were applied to understand how *S. aureus* is affected by metal ion starvation mimicking that imposed inside the host immune cells. The current study showed zinc deprivation significantly reduced *S. aureus* growth, biofilm formation, and hemolysis activity. Thus, zinc deprivation has a potential use in the supportive treatment of *S. aureus* topical infection. Results of this study can help in understanding the role of zinc metal ions in the pathogenesis of *S. aureus*, hence provide insight towards designing better preventive approaches targeting multiple facets of this process.

## 2. Results

### 2.1. Assessment of the Effect of Zinc Deprivation on Growth of S. aureus in Chemically Defined Media

**Figure 1:**
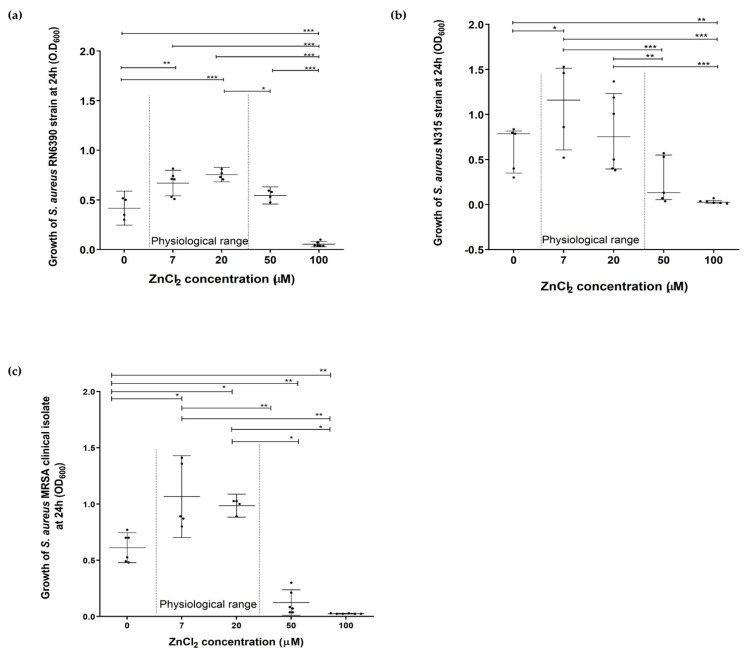
The effect of different zinc concentrations on the growth of *Staphylococcus aureus* at 24 h. The maximal effect of ZnCl_2_ deprivation was observed at 24 h compared to the physiological concentration range of ZnCl_2_ (7–20 µM). Zinc concentrations higher than 50 µM inhibited the growth significantly. (**a**) RN6390 strain, (**b**) N315 strain, and (**c**) methicillin-resistant *S. aureus* (MRSA) clinical isolate. Mann–Whitney test was used for statistical significance of the medians, where * *p* < 0.05, ** *p* < 0.01, *** *p* < 0.001. Data represented as medians with an interquartile range of at least four separate experiments.

The effects of zinc deprivation, high and toxic levels of zinc, 50 and 100 µM, respectively, were compared to the physiological range in humans (7 and 20 µM) on growth patterns of *S. aureus* RN6390, N315 strains, and MRSA clinical isolate [36,37]. Results showed that zinc deprivation led to a significant decrease in growth of all tested staphylococci at 24 h compared to the physiological range of zinc (7–20 µM) (*p* < 0.05) (Figure 1 and Supplementary Figure S1). High and toxic zinc levels 50 and 100 µM, respectively also inhibited *S. aureus* RN6390, N315 and MRSA clinical isolate growth at 24 h where the decrease was significant compared to physiological zinc levels (*p* < 0.05) (Figure 1 and Supplementary Figure S1). A way the bacterial population responds to adverse conditions to favor the survival of the community is dying and releasing nutrients from lysed cells. To test if this might be occurring, samples of *S. aureus* N315 strain cultivated in CDM with the corresponding zinc concentration were sampled at 0 and 24 h, respectively. The collected samples from each CDM at these specified time points were analyzed for their zinc content using ICP. The analysis revealed that bacterial inoculation into CDM with no zinc (ICP = 0.007 ppm) showed an increase in zinc content after 24 h incubation to become 4 µM (ICP = 0.564 ppm). Meanwhile, staphylococcal growth in CDM within the physiological range of zinc (ICP = 0.863 ppm equivalent to 6.33 µM) showed no significant change in zinc levels after 24 h incubation becoming 5.91 µM (ICP = 0.806 ppm).

### 2.2. Assessment of Effect of Zinc Deprivation on Biofilm Formation Ability of S. aureus in Chemically Defined Media Using Crystal Violet Microtiter Plate Method

**Figure 2:**
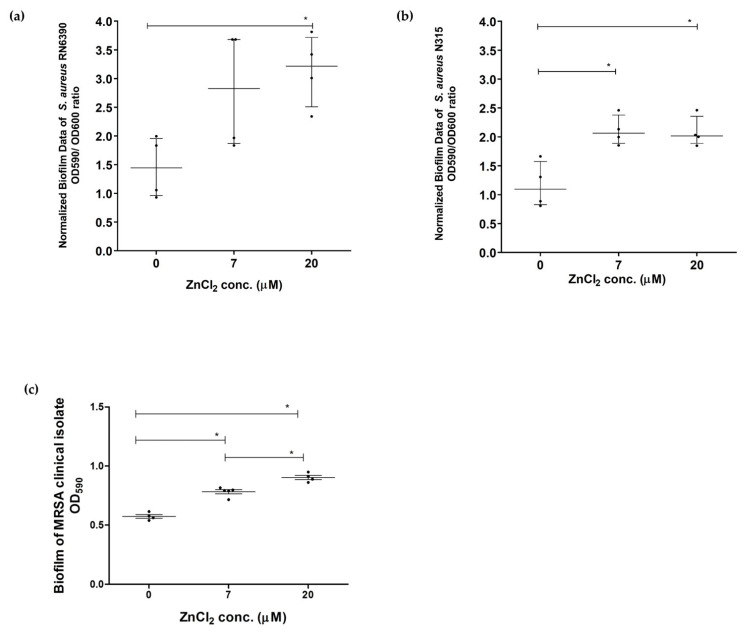
The ability of *S. aureus* tested strains to form biofilm in CDM with different zinc concentrations at 20 h. The ability of *S. aureus* (**a**) RN6390, (**b**) N315 strains, and (**c**) MRSA clinical isolate to form biofilm was significantly reduced due to zinc deprivation compared to 7 and 20 µM ZnCl_2_ (*p* < 0.05). Data represented as medians with an inter-quartile range of at least 4 separate experiments, biofilm data presented after normalization to growth. Mann–Whitney test was used to compare medians of the effect of zinc deprivation on biofilm formation, where * *p* < 0.05 was considered to be statistically significant.

The ability of *S. aureus* studied strains to form biofilm was preliminarily tested in CDM, BHI, and tryptone soya broth (TSB) media at different time points representing different biofilm stages. The effect of zinc deprivation was evident at 20 h, thus 20 h was chosen as a time point for further studies. Zinc deprivation significantly inhibited biofilm formation ability in *S. aureus* RN6390 compared to 20 µM zinc (*p* < 0.05) normalized to bacterial growth (Figure 2a). Similarly, zinc depletion significantly inhibited biofilm formation in *S. aureus* N315 compared to physiological range (7–20 µM) normalized to bacterial growth (*p* < 0.05) (Figure 2b) and MRSA clinical isolate (Figure 2c).

### 2.3. Assessment of the Effect of Zinc Deprivation on Oxidative Stress and Metalloproteases Activity of S. aureus Exemplified as Catalase Activity and Protease Activity

**Figure 3:**
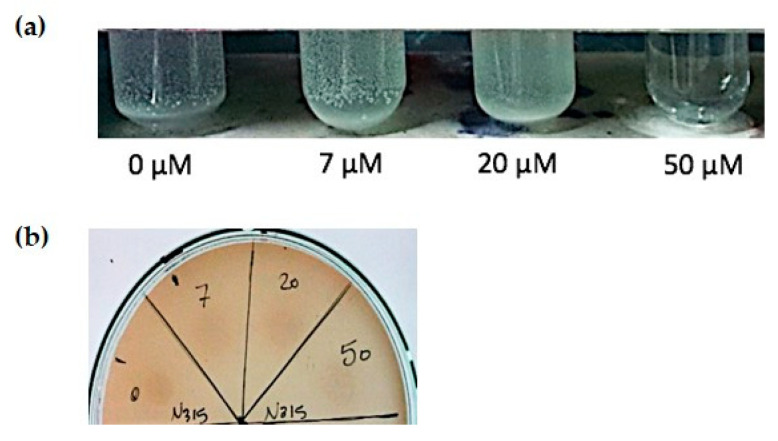
Assessment of catalase and protease activity of N315 *S. aureus* strain. (**a**) Catalase activity assessed in CDM, tubes were arranged according to zinc concentrations in CDM as follows; 0, 7, 20, and 50 µM. Better effervescence was observed at the physiological range of zinc (7–20 µM), while zinc limitation and high concentrations showed little or no effervescence, respectively. (**b**) Protease activity on skimmed milk agar of N315 *S. aureus* strain, cultures were grown in CDM with different zinc concentrations, no differences were observed.

The effect of zinc on catalase production was examined in overnight cultures of *S. aureus* N315. Catalase activity was measured as observed effervescence intensity after the addition of 3% hydrogen peroxide (H_2_O_2_) to overnight cultures in different zinc concentrations (0, 7, 20, 50, and 100 µM) CDM. Zinc deprivation showed less effervescence than that observed at the physiological range of zinc (7–20 µM) (Figure 3a). While bacterial cultures with zinc concentration 50 µM and higher showed no effervescence (Figure 3a). Next, metalloproteases activity of *S. aureus* N315 cultivated in CDM with different zinc concentrations on 3% skimmed milk agar was tested. N315 showed bacterial growth on the skimmed milk agar with no effect of zinc deprivation on protease activity (Figure 3b). Similar results were observed for RN6390 strain and MRSA clinical isolate (Figures S2 and S3).

### 2.4. Determination of the Effect of Zinc Deprivation on S. aureus Hemolytic Activity in Chemically Defined Media

Preliminary hemolytic activity was tested qualitatively on blood agar where *S. aureus* N315 showed gamma (γ) hemolysis at different zinc concentrations. To precisely examine the effect of zinc deprivation on the hemolytic activity of *S. aureus*, a quantitative assay for alpha-hemolytic activity was performed according to Bose J. et al. (2014) [40]. The hemolytic activity of *S. aureus* RN6390 grown under zinc deprivation was reduced even 50% hemolysis was not reached at starting concentration (no dilution) vs. bacteria grown at physiological zinc showed ~70% hemolysis (Table 1). N315 grown in zinc-deprived media showed 50% hemolysis at 2-fold dilution, while in the physiological range of zinc (20 µM), 50% hemolysis was achieved at a dilution of 6-fold (Table 2). This data indicated that *S. aureus* growing under zinc-deprived conditions showed lower hemolysis activity compared to those growing under physiological zinc range (20 µM) evident by requiring bacteria to be more concentrated for reaching 50% lysis. This might be due to the reduced expression of alpha-hemolysin. At a 16-fold dilution, N315 hemolytic activity was reduced by zinc deprivation (less than 10% hemolysis) compared to ~40% hemolysis for bacteria grown under 20 µM ZnCl_2_ conditions. In conclusion, the hemolytic activity of *S. aureus* RN6390 and N315 was reduced by zinc deprivation compared to 20 µM zinc.

### 2.5. Determination of the Effect of Zinc Deprivation on Differential Gene Expression of Selected Biofilm Associated Genes in S. aureus N315 under Biofilm Condition

**Figure 4:**
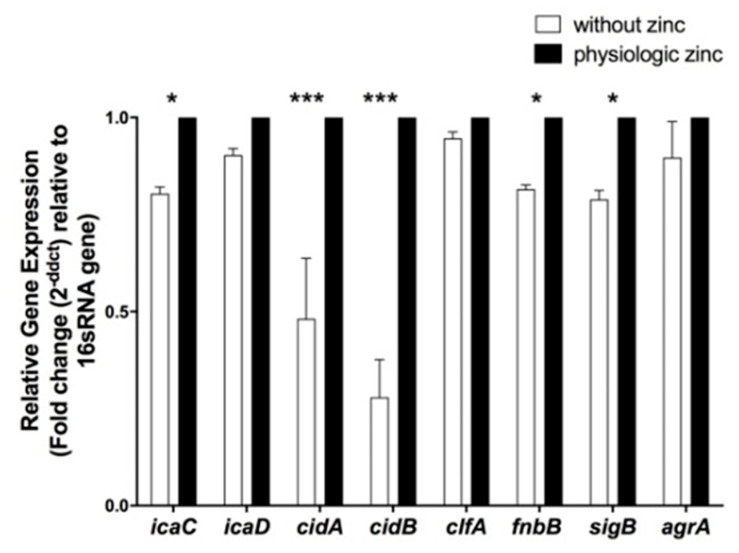
Effect of zinc limitation on differential gene expression of selected biofilm-associated genes in *S. aureus* standard strain N315 at 20 h under biofilm condition. Zinc limitation significantly downregulated adhesion molecule C (*icaC*) by 1.25 folds, murein hydrolase activator A and B (*cidA* and *B*) by 3 and 5 folds, respectively, fibronectin binding protein B (*fnbpB*) by 1.25 folds. Sigma factor B (*sigB*) was downregulated by 1.25 folds. Accessory gene regulator (*agrA*) and clumping factor A (*clfA*) showed no significant difference. Two-way ANOVA was used for statistical significance at * *p* < 0.05, *** *p* < 0.001. Data are represented as the average of at least three separate experiments.

To attempt to understand the genetic basis of biofilm formation was inhibited under zinc-depleted conditions, expression of biofilm-associated genes was examined in biofilm-forming N315 MRSA cells grown under zinc-deprivation at 20 h. Standard MRSA N315 strain was chosen for further molecular studies as it is a representative MRSA strain with complete genome sequence available, unlike MRSA clinical isolate. Moreover, MRSA is currently a bigger health problem than MSSA. Expression of the selected genes was presented as fold change normalized to16s ribosomal RNA (*16S rRNA*) gene, house-keeping gene normalizer, and relative to the expression to bacteria grown at 20 µM zinc (physiological conditions). Depletion of zinc significantly down-regulated the expression of genes associated with intercellular adhesion revealed that intercellular adhesion molecule C (*icaC*) by 1.25 folds while *icaD* expression level showed no significant change (Figure 4). Similarly, the depletion of zinc significantly downregulated the expression of genes associated with extracellular DNA (eDNA) production; murein hydrolase acti-vator A (*cidA*) and *cidB* by 3 and 5 folds, respectively (*p* < 0.05) (Figure 4). Furthermore, the gene associated with surface binding namely fibronectin binding protein B (*fnbpB*) was significantly downregulated by 1.25 folds, while clumping factor A (*clfA*) was not affected (Figure 4). The absence of zinc during the late stage of biofilm formation significantly downregulated the positive biofilm regulator sigma factor B (*sigB*) by 1.25 folds while the negative biofilm regulator accessory gene regulator (*agrA*) was not affected (Figure 4).

### 2.6. Determination of the Effect of Zinc Deprivation on Differential Gene Expression of Selected Protease and Hemolysis Associated Genes in S. aureus N315 under Planktonic Condition

**Figure 5:**
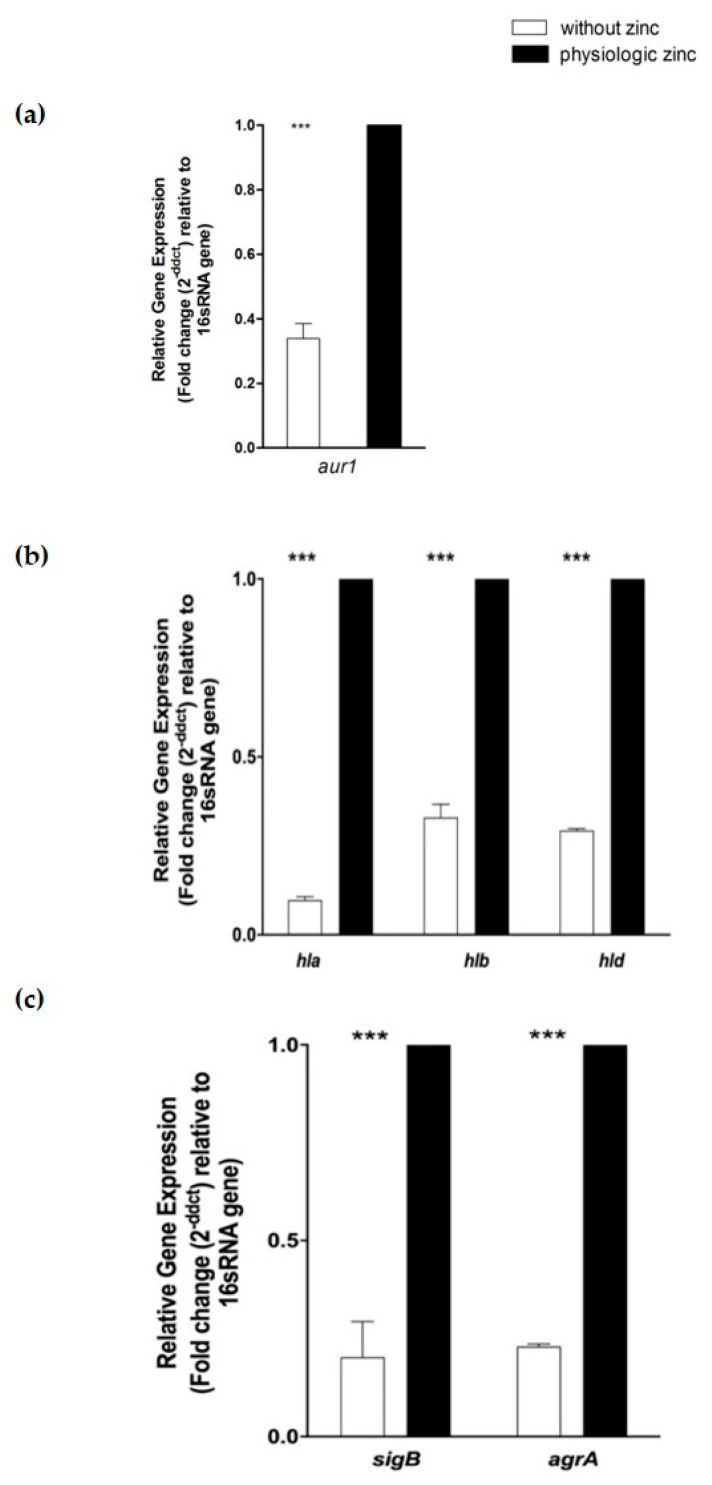
Effect of zinc limitation on differential gene expression of selected protease and hemolysis associated genes in *S. aureus* N315 under planktonic condition. At the late stage of growth (24 h), zinc limitation significantly down-regulated (**a**) zinc metalloprotease aureolysin (*aur1*) gene expression by 3 folds (*p* < 0.001), (**b**) alpha-hemolysin (*hla*), beta-hemolysin (*hlb*), delta-hemolysin (*hld*) by 10, 5 and 3 folds, respectively (*p* < 0.001), (**c**) *sigB* and *agrA* regulatory genes by 4.3 and 5 folds, respectively (*p* < 0.001). Two-way ANOVA was used for statistical significance at *** *p* < 0.001. Data are represented as the average of at least three separate experiments.

The levels of expression of selected protease- and hemolysis-associated genes were examined without zinc and at 20 µM zinc (physiological conditions) at 24 h under planktonic condition. In addition, genes encoding *sigB* and *agrA*, both parts of regulatory systems, were assayed for their expression under the same conditions. Differential gene expression analysis showed that zinc deprivation significantly down-regulated zinc metalloprotease aureolysin (*aur1*) gene associated with protease activity (*p* < 0.001) by 3 folds (Figure 5a). In addition, zinc deprivation significantly down-regulated hemolysis associated genes; alpha-hemolysin (*hla)*, beta-hemolysin (*hlb*), and delta-hemolysin (*hld*) by 10, 5, and 3 folds respectively (*p* < 0.001) (Figure 5b). Regulatory genes *sigB* and *agrA* were significantly downregulated by 4.3 and 5 folds, respectively, upon zinc deprivation (Figure 5c).

## 3. Discussion

*Staphylococcus aureus* remarkably resists many antibiotics from methicillin (MRSA) to vancomycin (VRSA). The emergence of antibiotic-resistant *S. aureus* strains creates a persistent need for developing new therapeutics for staphylococcal-associated diseases [41]. A promising approach to combat staphylococcal infections is by disrupting its ability to overcome the host immune response, this can be through the use of metal limitation [33]. Among the important metals that play a relevant role during host colonization by bacteria is zinc. Zinc is the second most abundant transition metal cofactor after iron; it is required for many metabolic processes [42]. The importance of zinc metal ion for *S. aureus* can be targeted as a new weapon for combating staphylococcal infections by limiting its availability. Although it is known that vertebrates sequester zinc, the extent of metal starvation imposed on *S. aureus* is not well-studied [43]. 

In the current study, the effect of zinc deprivation on the growth and selected virulence factors of *S. aureus* N315 and RN6390 strains was studied. To properly study the effect of zinc deprivation, it was crucial to prepare a chemically defined medium (CDM) containing all metals except zinc and another CDM containing the physiologic zinc concentrations. Several studies showed zinc reference ranges needed for homeostasis in the human body in different organs. Studies have shown that physiologically relevant zinc levels encountered by the bacteria in the human body ranged between 7 and 20 µM [35,36,37,38,39]. Studies showed that zinc concentrations below 7 µM had no significant rescuing effect on *S. aureus* [31,34]. Thus, the used zinc levels in CDM with zinc were chosen to mimic the physiologically relevant concentrations occurring in the human body (7 and 20 µM). CDM without zinc was used to mimic the conditions imposed by the human immune cells on bacteria, where calprotectin and other proteins subject bacteria to complete deprivation of zinc [15,25]. Thus, zinc deprivation was explored in the current study as a possible approach to combating *S. aureus* infections.

The zinc levels in the prepared CDM without zinc were estimated by zincon colorimetric assay [44]. The zincon assay revealed minute levels of zinc in the prepared CDM using a calibration curve. For accurate determination of zinc concentrations in the prepared CDM with specified zinc concentrations (absence, 7 and 20 µM), the highly sensitive inductively coupled plasma spectrometry (ICP) method was used. ICP is an analytical technique developed to detect elements with atomic mass ranges 7 to 250 available at very low concentrations [35,45]. ICP technology is capable of detecting most of the periodic table of elements at milligram to nanogram levels per liter. Zinc concentration as little as 0.04 ppm could be detected at a wavelength of 481.053 nm [46]. ICP showed that the prepared CDM at the specified zinc concentrations were acceptable (Supplementary Table S1). Based on our results, zincon colorimetric assay was not reliable to assess zinc concentrations, only ICP-MS would be recommended to be used for accurate determination of zinc concentrations.

Bacterial cultures of *S. aureus* N315 grown at 0 and 24 h in CDM at specified zinc concentrations were tested for changes in zinc content. The results showed that in the case of CDM with no zinc, bacterial growth led to zinc release in the external environment, while bacteria growing in culture media with physiologic zinc concentrations showed no change. This could be due to the released zinc from dead bacteria into the CDM. Our study showed that zinc at 7–20 µM was required for *S. aureus* growth similar to Lindsay and Foster (2001) study where the addition of 20 µM zinc enhanced the growth of *S. aureus* 8325-4 in a chemically limited (CL) medium. Lindsay and Foster (2001) showed that growth enhancement is mediated by two ABC transporters, mreA and mreB [31]. Additional studies on other MRSA strains should prove useful as those studies we performed on MRSA isolate showed similar results to those of the N315 strain. Therefore, future studies on other MRSA standard strains would add emphasis to our current findings observed on the widely studied N315 MRSA strain.

**Figure 6:**
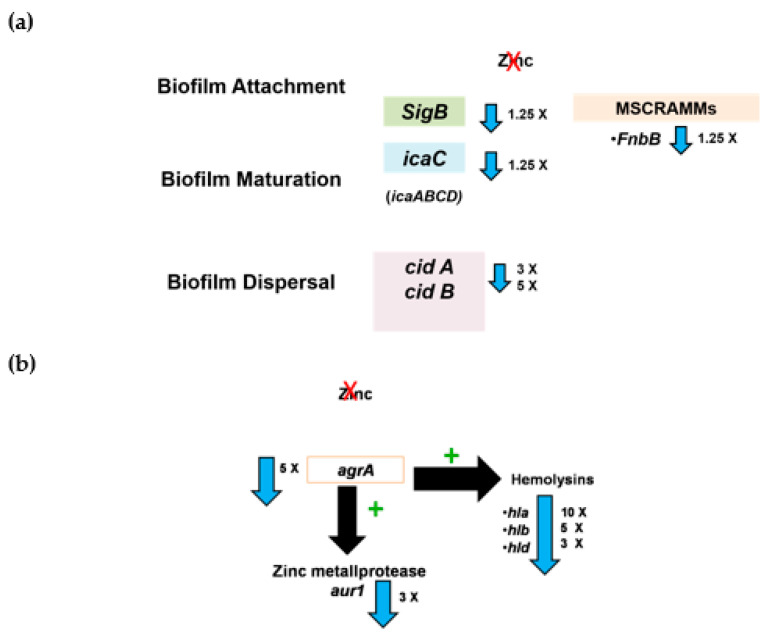
Regulatory factors controlling biofilm and hemolysins in *S. aureus* and how zinc deprivation affected their gene expression. (**a**) Several regulators control biofilm formation; extracellular matrix adhesive molecules required for adhesion are negatively regulated by the quorum-sensing regulator *agrA* while PIA encoding genes are upregulated by *sig B* while *agrA* has no regulatory role in PIA expression. In the current study, gene expression analysis of selected biofilm-associated genes revealed that *fnbpB* was downregulated by 1.25 folds. *IcaC* only (PIA exporter) was down-regulated upon zinc limitation by 1.25 folds. The genes that regulate eDNA formation; *cidA* and *cid B* were downregulated by 3 and 5 folds, respectively; (**b**) Gene expression analysis showed that absence of zinc significantly downregulated expression of *agrA*; the prototype quorum-sensing regulator gene by 5 folds (*p* < 0.001). Accordingly, the effect of zinc limitation on the expression level of the Agr-regulated toxins; alpha-hemolysin, delta- hemolysin, and zinc metalloprotease was tested at 24 h at planktonic growth conditions. The absence of zinc significantly downregulated expression of the hemolysins where *hla* was downregulated by 10 folds, *hlb* by 5 folds, and *hld* by 3 folds compared to physiological zinc concentration (*p* < 0.001). The expression level of the zinc-associated metalloproteinase aureolysin *aur1* was significantly reduced by 3 folds (*p* < 0.001).

*Staphylococcus aureus* N315 biofilm at later stages (20 h) was significantly inhibited by zinc deprivation compared to physiologic zinc levels (7–20 µM). Gene expression analysis revealed that zinc depletion affected the expression of genes associated with biofilm attachment (*fnbpB*) and maturation (*icaC* gene part of icaADBC-encoded polysaccharide intercellular adhesin (PIA)) and extracellular DNA (eDNA) dispersal (*cidA* and *cidB*) (summarized in Figure 6a). *fnbpB* encodes a fibrinogen-binding protein, a prominent member of microbial surface components recognizing adhesive matrix molecules (MSCRAMMs). MSCRAMMs mediate attachment to indwelling devices, bacteria-host adhesion, and bacterial colonization [11,47]. This suggested that zinc depletion affected several steps in biofilm formation, attachment, and maturation in a PIA-dependent manner [48]. Moreover, *sigB* gene encoding sigma factor B, a regulator of biofilm, was also downregulated. Our finding that zinc deprivation inhibited biofilm formation is similar to previous reports showing that zinc is important for biofilm formation in *S. aureus* USA300 and SH1000 strains [32,49,50]. Likewise, a derivative of the compound 2-aminobenzimidazole (2-ABI), directly bound to ZnCl_2_ was found to act as a biofilm inhibitor of MRSA, VRSA, and *S. epidermidis* biofilms in zinc-dependent mechanism [51]. However, previous studies on zinc deprivation effect on *S. aureus* biofilm used microbiological media like brain-heart infusion or tryptic soy agar with metal chelators. Here, we used a chemically defined medium to better study the effect of zinc deprivation on biofilm formation in a controlled approach in the presence of other metals. Chemically defined media are of value in studying the minimal nutritional requirements of microorganisms and provide a basis to investigate the requirements of the microbial cell [52]. Therefore, the use of chemically defined media is a must to be able to govern the concentration and hence the availability of zinc in minute amounts mimicking the physiologically relevant concentrations occurring in the human body the organism encounters within the immune system.

*Staphylococcus aureus* genome encodes three families of proteases: metalloprotease, cysteine, and serine proteases [53]. The metalloproteinase: aureolysin (Aur) is also known as protease III and is the only metalloprotease produced by *S. aureus* which requires zinc ions for its activity. Aureolysin is activated by auto-proteolytic cleavage initiating a proteolytic cascade of activation of other proteases [54]. The metalloprotease aureolysin plays an important role in staphylococcal inhibition of the complement system and helps *S. aureus* in immune system evasion through mediating the cleavage of the antimicrobial peptide LL-37 [55,56]. In addition, it contributes to bacterial spread and invasion via activation of the human fibrinolytic system [55]. The important role of zinc metalloproteinase aureolysin in staphylococcal pathogenesis requires further investigation of the impact of zinc sequestering effect on its activity. In the current study, the effect of zinc limitation on protease activity was examined qualitatively on skimmed milk agar plates. This assay could not report the effect of zinc limitation on protease activity in *S. aureus*. This required further assessment by studying the aureolysin gene expression and the expression of its regulators. Aureolysin is the only metalloprotease produced by *S. aureus* and requires zinc ions for its activity [54]. This might provide an explanation for the downregulation of aureolysin encoding gene (*aur*) in the absence of zinc as it has been reported that the activity of aureolysin was inhibited by the metal chelator EDTA, the calcium chelator EGTA and the zinc-specific chelator 1,10-phenanthroline [57]. Two central regulators (accessory gene regulator (*agr*) and *sigB*) of metalloproteases operon and cytotoxins were down-regulated suggesting an effect of zinc deprivation on several proteases and hemolysins [58] (summarized in Figure 6b). 

*Staphylococcus aureus* secretes a variety of interacting toxins affecting the plasma membrane of the host cells including human erythrocytes [59]. Our study revealed that zinc deprivation significantly inhibited hemolytic activity of *S. aureus* N315 and RN6390 and downregulated expression levels of *hla*, *hlb*, and *hld* genes encoding alpha, beta, and delta hemolysins of *S. aureus* respectively (Figure 6b). Although alpha-hemolysin is one of the most studied of *S. aureus* cytotoxins [60], there are no studies, to the best of our knowledge, investigating the role of zinc deprivation on *S. aureus* hemolysins expression and activity. The most relevant study was on the mechanism of Hla-mediated cell lysis via binding to A-disintegrin and metalloprotease 10 (ADAM10); a zinc-dependent metalloprotease trans-membrane protein on human cells. Consequently, it was postulated by Wilke and Wardenburg that the use of zinc metalloprotease inhibitors including ADAM10 might be useful in combating staphylococcal infections [61]. Previous studies suggested a possible impact of zinc on hemolysins activity in different pathogens other than *S. aureus*. For instance, *Clostridium perfringens* and *E. coli* hemolysins induce cell leakage of certain metabolites through stable pore formation in the cells. This leakage was inhibited by the addition of zinc, calcium, and magnesium divalent cations [62,63]. Moreover, Velasco et al. (2018) were the first to describe the involvement of zinc deprivation and zinc uptake regulator (zur) in the expression of an alpha-hemolysin virulence factor in clinical isolates of uropathogenic *E. coli* [29]. On the contrary, zur has no apparent role in staphylococcal pathogenicity examined using a mouse-abscess model [31]. 

Studies have shown that zinc starvation can be imposed by the host during infection and is mediated by zinc-binding proteins e.g., calprotectin and metallothionein limiting bacterial viability and virulence [25,26,27]. The feasibility of limiting metal ions in the treatment and prevention of infection has been explored in the use of lactoferrin, which is an iron-binding glycoprotein, in the supportive treatment of *H. pylori* infections [24], prevention of antibiotic-associated diarrhea and *Clostridium difficile* infections [64,65] and ulcerative colitis [66]. Moreover, antimicrobial peptides (AMP) as lactoferrin have been used as a coating for dental, bone, and other medically used implants [67]. Therefore, to apply the findings of this study in clinical settings, we propose the use of either zinc-specific chelating agents e.g., 1,10-phenanthroline or diethylenetriaminepentaacetic acid, or recombinant AMP as recombinant calprotectin or metallothionein in the supportive treatment of topical infection. Concerns regarding the effect of zinc deprivation on human cells has been studied previously where human keratinocytes (HaCaT cells) exposed to zinc deficiency induced by TPEN a high-affinity zinc chelator showed no changes in cell viability and growth, or in the cytoskeletal and cell adhesion systems over a period of 7 days [68]. The current study in vitro results suggested that zinc deprivation for 24 to 48 h might be effective as a supportive treatment in topical *S. aureus* infections. Thus, the duration of zinc deprivation is less than 7 days, after which the induction of apoptosis can be observed [68]. Zinc chelators thus might be for example incorporated in the wound dressings for infected wounds or as a coating for medical devices and implants or as a component of rinses in surgeries around the area of the implant. 

## 4. Materials and Methods

### 4.1. Bacterial Strains Maintenance and Culturing Conditions

RN6390 and N315 strains were used as standard methicillin-sensitive *S. aureus* (MSSA) and methicillin-resistant *S. aureus* (MRSA) strains, respectively, both are well-defined standard strains [69,70]. N315 strain and the used MRSA clinical isolate are both mec-A gene-positive MRSA [70,71]. Moreover, the N315 strain was chosen for further molecular studies as it is a representative MRSA with a complete genome sequence available. Standard strains confirmatory identifications were performed including gram staining, biochemical testing, and antibiotic susceptibility testing by Kirby-Bauer disk diffusion method using methicillin (5 µg), cefoxitin (30 µg), and oxacillin (1 µg) disks [72]. Fresh cultures of RN6390, N315 strains, and MRSA clinical isolate were cultured from −80 °C glycerol stocks by streaking on brain heart infusion (BHI) agar and incubated overnight at 37 °C. Sub-cultures were prepared by transferring three to five colonies into 5 mL of chemically defined medium (CDM) in sterile glass 15 mL tubes and incubated overnight at 37 °C, shaking at 180 rpm. The optical density at 600 nm (OD600nm) of the overnight cultures was adjusted by appropriate dilution with respective medium to 1, followed by 50 times dilution in the media with the corresponding ZnCl_2_ concentrations (0, 7, 20, 50 µM) in sterile glass test tubes. Then the cultures were incubated at 37 °C and 180 rpm and used for relevant experiments or assay. The clinical MRSA isolate was from a collaborator and was identified by conventional biochemical testing including yellow colonies on mannitol salt agar base, positive coagulase, catalase, and typical *S. aureus* oxidation-fermentation reactions. Moreover, biochemical testing using an API kit confirmed the isolate to be *S. aureus*. Antimicrobial susceptibility testing of the MRSA clinical isolate showed that it was resistant to oxacillin, methicillin, and cefoxitin and sensitive to vancomycin and linezolid. Moreover, oxacillin resistance screening agar base (ORSAB) showed blue colonies indicative of oxacillin resistance [71].

### 4.2. Chemically Defined Medium for Assessment of Zinc Deprivation

Time and effort were put into preparing CDM and troubleshooting the preparation till we finally used modified Vitko and Richardson (2013) [73]. The amounts and order of addition of the medium components in specific the salts and trace elements solutions were according to Taylor and Holland (1989) and Hussain et al. (1991) [30,74] (Table 3). In brief for salt solution preparation, Na_2_HPO_4_, K_2_HPO_4_, (NH_4_)_2_SO_4_, and MgSO_4_.7H_2_O were dissolved in deionized water while MgSO_4_.7H_2_O solution was prepared separately as it caused precipitation in the salt solution. For amino acids solutions, each phenylalanine and isoleucine was dissolved separately in 1 M NH_4_OH. Proline, valine, glycine, threonine, alanine, and serine each was dissolved in deionized water, while the rest of the amino acids were dissolved each separately in 1 N HCl. Each of the bases (U, C, G, A, T) was dissolved separately in deionized water. The vitamins solution was prepared as a stock solution containing thiamine, niacin, biotin, and calcium pantothenate dissolved together in deionized water. All the previous solutions were sterilized individually by autoclaving, while the trace metal element solution was sterilized by filtration. Various trace element solutions were incorporated in the chemically defined medium to test the interactions between nickel, cobalt, and zinc as stated by Remy et al. (2012) [75]. After sterilization, each culture medium was constituted by adding 2 mL of the salt solution, 1 mL of each amino acid, 1 mL of each base, 0.1 mL of the vitamin solution, and 0.1 mL of the trace metal elements solution, 4 mL of 10% glucose solution as a carbon source, and finally MgSO_4_.7H_2_O to the beaker while slowly stirring the mixture. The pH of the medium was then adjusted to 7.4 using 10 M NaOH, and the final volume of the medium was brought up to 100 mL using deionized water then the reconstituted medium was filter sterilized.

Zinc concentrations in the CDM containing zinc were chosen to match physiologically relevant levels encountered by bacteria in the human body between 7 and 20 µM [36,37]. In the CDM without zinc, the environment of the immune cells where calprotectin among other proteins completely deprives the bacteria of zinc was mimicked, thus the choice of no zinc in the prepared CDM without zinc.

### 4.3. Analysis of Zinc Concentrations in the Prepared Chemically Defined Media

Two analytical approaches were used to estimate zinc levels in the prepared CDM; zincon assay and inductively coupled plasma spectrometry (ICP). 

#### 4.3.1. Zincon Assay 

Colorimetric determination of zinc using zincon assay following Platte and Marcy (1959) method as an analytical estimate of zinc [44]. The assay depends on the use of zincon (2-carboxy-2′-hydroxy-5′-sulfoformazylbenzene) that can form a blue zinc–zincon complex with zinc metal ion. A calibration curve obtained from known amounts of zinc was constructed and it showed the minute amount of zinc in the CDM without zinc (calculated from the calibration curve Supplementary Figure S2).

#### 4.3.2. Inductively Coupled Plasma Spectrometry (ICP)

The second approach for determining zinc levels was the advanced analytical technique of ICP, a highly sensitive analytical technique developed to detect elements available at very low concentrations [35,45]. Samples were analyzed at the Faculty of Agriculture, Ain Shams University using Profile Plus high dispersion ICP, USA. Zinc concentration as little as 0.04 ppm could be detected at a wavelength of 481.053 nm. The amount of ZnCl_2_ in ppm can be calculated as follows:

(1) $$1{\;µM\;\mathrm{ZnCl}}_{2}\equiv 136.286\times 10^{-6}\;g\;/{\;L\;\mathrm{ZnCl}}_{2}\;\equiv 0.136286{\;\mathrm{ppm}\;\mathrm{ZnCl}}_{2}$$

ICP confirmed that each of the prepared CDM was containing the intended amount of zinc (in the form of ZnCl_2_) as shown in Supplementary Table S1.

### 4.4. Determination of the Effect of Zinc Deprivation on the Growth of Selected S. aureus Strains Using Chemically Defined Media

Cultures were prepared as detailed under bacterial strains maintenance and culturing conditions and the OD_600nm_ was measured at different time intervals 6, 12, 24, 48 h to choose the suitable time point that better show the effect of zinc limitation on staphylococcal growth.

### 4.5. Determination of Effect of Zinc Deprivation on the Biofilm Formation of Selected S. aureus Strains Using Crystal Violet Microtiter Plate Assay Method

To test the biofilm-forming ability of *S. aureus* in CDM, cultures were prepared as detailed under bacterial strains maintenance and culturing conditions then, 200 µL of the diluted samples were added into the wells of a sterile 96 well non-pyrogenic polystyrene culture plate tested as quadrates (four technical replica) for each sample. Chemically defined medium with no zinc represented the negative control. The plates were incubated at 37 °C statically for 12 and 24 h. On the second day, planktonic growth was measured at 600 nm using 96-well plate reader Synergy 2 then the solution was discarded, and the wells were washed with PBS three times and the plate was left to fully dry. Crystal violet was prepared as 0.5% in deionized water and left in a shaker incubator at 37 °C overnight for complete dissolution. For visualizing the adherent cells, 200 µL of 0.5% crystal violet were added to each well and the plate was left static for 30 min. The plate was then washed with sterile deionized water three times then left to completely dry. To measure the optical density of the stained cells, 200 µL of 96% ethanol were added to each well, and the plate was put onto a shaker at 115 rpm for 15 min, then 125 µL were transferred to a new plate. The absorbance of the transferred stained cells was measured at 590 nm using 96-wells plate reader Synergy 2. The obtained data were interpreted using the following formula to determine the extent of biofilm formation ability in each organism, BF = AB − CW, where BF is the biofilm formation, AB is the OD590 nm of stained attached bacteria and CW is the OD590 nm of stained control wells containing bacteria-free medium. The ratio of the OD_590_/OD_600_ was used to normalize the amount of biofilm formed to the growth of bacteria in the presence and absence of different ZnCl_2_ concentrations [76].

### 4.6. Qualitative Assessment of the Effect of Zinc Deprivation on the Oxidative Stress Exemplified as Catalase Activity of Selected S. aureus Strains in Chemically Defined Media

Cultures were prepared as detailed under bacterial strains maintenance and culturing conditions and the overnight cultures were centrifuged at 13,000 rpm for 5 min then the supernatant was tested for catalase activity by adding 1 mL of 3% H_2_O_2_ to 5 mL of bacterial supernatant of the corresponding zinc concentration and the intensity of effervescence formation was observed [77].

### 4.7. Qualitative Assessment of the Effect of Zinc Deprivation on the Metalloproteases Exemplified as Protease Activity of Selected S. aureus Strains in Chemically Defined Media

Cultures were prepared as detailed under bacterial strains maintenance and culturing conditions and then 10 µL of the adjusted bacterial culture was spotted on 3% Skimmed milk agar, respectively. The spotted cultures were allowed to dry aseptically and incubated at 37 °C, 24 h. After 24 h incubation, the plates were examined for the presence of transparent zones around the spots or the growth of bacteria from the inoculation site or halo formation. Some bacterial cultures needed further 24 h incubation at 4 °C for better visualization of the protease activity [78]. 

### 4.8. Quantitative Assessment of the Effect of Zinc Deprivation on the Hemolytic Activity of Selected S. aureus Strains in Chemically Defined Media

Cultures were prepared as detailed under bacterial strains maintenance and culturing conditions and then 10 µL of each diluted bacterial culture were spotted on blood agar to be incubated overnight at 37 °C. Complete hemolysis appeared as a clear zone around the bacterial growth [56,79].

A quantitative assessment of the effect of zinc depletion on the alpha-hemolytic activity of *S. aureus* assay was performed as described elsewhere with some modifications [40,80]. Briefly, a single colony was used to inoculate 5 mL of CDM with different zinc concentrations. The cultures were incubated at 37 °C with shaking at 180 rpm for 24 h then the samples were placed on ice. The cultures were diluted with the corresponding CDM to equalize the OD_600nm_ to a value within 0.05, pelleted by centrifugation at 4 °C, and sterile filtered through a 0.2 µm filter. Serial 2-fold dilutions of each bacteria-free supernatant in PBS were incubated for 1 h at 37 °C with a ~2% to 4% solution of PBS-washed human red blood corpuscles in Alsever’s solution. The heme-containing supernatant was harvested by centrifugation and was assessed by measuring absorbance at 450 nm. The dilution required to achieve maximal lysis was compared to complete lysis with 0.1% SDS control (positive or total lysis control) and untreated RBCs in buffer solution (blank or negative control) at each CDM with different zinc concentrations. Percent hemolysis was calculated based on the equation by Costabile M. (2010) [81] as follows:

(2) $$\mathrm{Percent}\;\mathrm{hemolysis}\;\left(\%\right)=\;\frac{{\mathrm{OD}}_{450}\;(\mathrm{test})\;-{\mathrm{OD}}_{450}\;(\mathrm{blank})}{{\mathrm{OD}}_{450}\;\left(\mathrm{total}\;\mathrm{lysis}\right)\;-{\mathrm{OD}}_{450}\;(\mathrm{blank})}\;\times \;100$$

### 4.9. Determination of the Effect of Zinc Deprivation on Differential Gene Expression of Selected Genes in S. aureus N315 under Planktonic and Biofilm Conditions

#### 4.9.1. Total RNA Isolation

*Staphylococcus aureus* N315 cultures were prepared as detailed under bacterial strains maintenance and culturing conditions however in a 24-well plate instead of 15 mL glass tubes. A volume of 5 mL of each adjusted culture in CDM with its corresponding zinc concentration was incubated at 37 °C and 180 rpm for 24 h for planktonic growth. While a volume of 2 mL of each CDM was added into 24-well plates which were incubated statically for 20 h at 37 °C for biofilm growth. Planktonic cells grown for 24 h were prepared for RNA isolation by being pelleted by centrifugation at 4000 rpm for 20 min. The supernatant was discarded, and the pelleted cells were collected to proceed to the total RNA isolation.

Biofilm forming cells adhering to the surface of each well after 20 h were after the planktonic cells were washed off by 2 mL of PBS (pH = 7.4) for two consecutive times. The biofilm-forming (adherent) cells were collected into 1.5 mL sterile RNase-DNase free tubes by washing cells with PBS after scrapping, followed by three alternating times of sonication for 1 min each and intermittent incubation on ice for 30 s. The cells were pelleted by centrifugation at 13,000 rpm for 2 min and the PBS was discarded.

Total RNA was isolated according to the manufacturer’s protocol of Direct-zol TM RNA miniprep (Zymo Research, Irvine, CA, USA). Briefly, the bacterial culture pellets were resuspended in 600 µL trizol reagent, mixed well by gentle vortexing for 30 s then was transferred into NucleoSpin bead tube type B for cell lysis (Macherey-Nagel, Düren, Germany). Cells were lysed by vortexing the beads columns at a speed of 2700 rpm for 5 min using Vortex-Genie 2. This step was repeated four times with intermittent incubation on ice for 60 s for a total of 20 min. Then the beads column was spined at 13,000 rpm for 2 min followed by transferring 570 µL of the supernatant to a sterile 1.5 mL tube without disturbing the pellet then proceeding according to manufacturer protocol including DNA removal. The concentration and purity of the isolated RNA were evaluated using UV-Visible Spectro Nanophotometer (IMPLEN GmbH, Munich, Germany), the integrity of the RNA was evaluated by running on 0.8% agarose gel, and 16 s and 23 s rRNA bands were visualized.

#### 4.9.2. Complementary DNA (cDNA) Synthesis

Complementary DNA (cDNA) was prepared according to Quantitect reverse transcription kit protocol (Qiagen, Hilden, Germany). The concentration and purity of the resultant cDNA were evaluated using UV-Visible Spectro Nanophotometer. The prepared cDNA was diluted ten times, concentrations were measured again, and then the diluted cDNA was stored at −20 °C. Two negative controls were included a no template control (NTC), a full reaction without RNA template to serve as a general control for extraneous nucleic acid contamination. The second control was a no reverse transcriptase control (NRT) or minus reverse transcriptase control (-RT).

#### 4.9.3. Quantitative Real-Time PCR Analysis of Differentially Expressed Genes

The changes in gene expression due to zinc deprivation were quantified by Real Time-PCR (qPCR) using Sensifast SYBR Green PCR Kit (Bioline, London, UK) on a Rotor-Gene Real-Time PCR machine (Qiagen, Hilden, Germany). Primers were designed using the integrated DNA technologies (IDT) primer quest tool (Integrated DNA Technologies, Coralville, IA, USA) except for the 16s rRNA gene primer that was based on Attia et al. (2010) [82]. Primers sequences, annealing temperatures, and expected amplicon size are shown in Table 4. Primers were manufactured by IDT and Macrogen Inc., (Seoul, Korea). The thermal cycling conditions were optimized as follows: initial activation at 95 °C for 2 min; followed by 35 cycles of denaturation at 95 °C for 5 s; annealing at 55–60 °C for 10 s; extension at 72 °C for 20 s followed by data acquisition after each cycle. All PCR tests were carried out in duplicates of each treatment from three different biological replica experiments. Expression of selected genes was presented as fold change normalized to 16s rRNA gene, house-keeping gene normalizer, and relative to the expression at 20 µM zinc. 

### 4.10. Statistical Analysis 

Data were plotted and analyzed using GraphPad Prism 5.01 (GraphPad Software Inc., San Diego, CA, USA). The results of zinc deprivation effect on growth and biofilm formation of *S. aureus* were graphically presented as medians with an inter-quartile range of at least four independent experiments. One-way ANOVA analysis was used to compare the effect of different concentrations of zinc on the growth of *S. aureus*, followed by Tukey’s multiple comparison test to compare the replicate means, where *p* < 0.05 was considered to be statistically significant. Mann–Whitney test was used to compare medians of the effect of zinc deprivation on biofilm formation, where *p* < 0.05 was considered to be statistically significant. The results of gene expression analysis were presented as means ± standard deviation of means representative of three independent experiments. Two-way ANOVA analysis was used to compare the effect of zinc deprivation on the gene expression levels, followed by Bonferroni post hoc test to compare the replicate means, where *p* < 0.05 was considered to be statistically significant.

## 5. Conclusions

There is an urgent need for developing new strategies that can overcome the continuous ability of staphylococcus to acquire resistance against the commonly used antimicrobial agents. An alternative approach is to develop anti-virulence therapies that interfere with bacterial toxins or virulence factors or pathways that regulate toxins or virulence factors production [83]. The current pilot study focused on exploring the effect of zinc deprivation on the expression level of genes involved in hemolysins, protease, and biofilm regulation in *S. aureus* N315 standard strain. This is a pilot study, therefore, a few numbers of strains were tested to merely explore the possible effect of zinc deprivation on *S. aureus*. However, testing more *S. aureus* strains and/or clinical isolates should prove beneficial towards a deeper understanding of the effect of zinc starvation on the pathogenesis of *S. aureus*. Our study provides evidence that the hemolytic activity of *S. aureus* is affected by zinc depletion when assayed phenotypically and on the genetic level. To the best of our knowledge, our study is the first to report a direct effect of zinc deprivation on hemolysins gene expression in *the S. aureus* N315 strain. These results emphasize the importance of targeting *S. aureus* secreted toxins and the probability of anti-virulence therapies being used as new approaches in combating staphylococcal infections and preventing harmful effects imposed by these toxins to host cells. In addition, this study suggests a new insight into the utilization of zinc as a factor that might help in limiting biofilm-associated infections. Exploring the competition for zinc between *S. aureus* and the highly zinc adsorbing fungus *Rhizopus arrhizus* [84] can be applied in thwarting biofilm formation on indwelling devices. Moreover, the use of either zinc-specific chelating agents e.g., 1,10-phenanthroline or recombinant calprotectin or metallothionein (natural antimicrobial peptides that chelate zinc) can prove useful in facing microbial local colonization thus limiting systemic dissemination. These zinc chelators can be used in the form of wound dressings for infected wounds or applied as a coating for medical devices and implants or as an ingredient in surgical rinses to prevent the spread of topical staphylococcal infections.

## Figures and Tables

**Table 1:** Quantitative assessment of hemolytic activity of *Staphylococcus aureus* RN6390 in chemically defined media under the effect of zinc deprivation.

Dilution	Zinc Depleted Conditions		Physiological Zinc (20 µM zinc)	
	Mean Absorbance at 450 nm	Mean Percent Hemolysis (%) *	Mean Absorbance at 450 nm	Mean Percent Hemolysis (%)
1 **	0.969	44.09	1.389	67.492
(1:2)	0.66	26.89	0.71	29.565
(1:4)	0.51	18.318	0.61	23.94
(1:8)	0.38	11.19	0.39	11.8
(1:16)	0.34	9.24	0.35	9.688
(1:32)	0.2	1.22	0.38	11.19

* positive control using 0.1% sodium dodecyl sulfate (SDS) showed complete hemolysis of human red blood corpuscles and negative control was untreated human red blood corpuscles in buffer solution. ** Zinc deprivation reduced the hemolytic activity of *S. aureus* RN6390 significantly (*p* < 0.0001) compared to 20 µM.

**Table 2:** Quantitative assessment of hemolytic activity of *S. aureus* N315 in chemically defined media under the effect of zinc deprivation.

Dilution	Zinc Depleted Conditions		Physiological Zinc (20 µM zinc)	
	Mean Absorbance at 450 nm	Mean Percent Hemolysis (%) *	Mean Absorbance at 450 nm	Mean Percent Hemolysis (%)
1	1.342	77.17	1.43	80.79
(1:2)	0.87	46.88	1.12	62.639
(1:4)	0.8	42.817	0.95	52.817
(1:8)	0.34	12.416	0.76	37.527
(1:16) **	0.24	7.01	0.668	34.3
(1:32)	0.27	6.06	0.35	13.752

* positive control using 0.1% SDS showed complete hemolysis of human red blood corpuscles and negative control was untreated human red blood corpuscles in buffer solution. ** significantly reduced hemolytic activity (*p* < 0.0001) compared to 20 µM.

**Table 3:** Chemically Defined Medium components and concentrations.

Component	Concentration
**Salt solution**	
Ammonium sulfate ≥ 99%	2 g/L
Magnesium sulfate heptahydrate	0.5 g/L
Potassium phosphate monobasic	4 g/L
Sodium phosphate dibasic	4 g/L
**Amino acid solution**	
L (+) Glutamic acid 99%	10 g/L
L (+)-Aspartic acid, 98+%	9 g/L
L-Alanine	6 g/L
L-Arginine	7 g/L
L-Cystine	2 g/L
L-Histidine	3 g/L
L-Isoleucine	3 g/L
L-Leucine	9 g/L
L-Lysine monohydrochloride	1 g/L
L-Methionine	7 g/L
L-Phenylalanine	5 g/L
L-Proline 99%	1 g/L
L-Serine for chemistry	3 g/L
L-Threonine	3 g/L
L-Tryptophan	1 g/L
L-Tyrosine 99+%	5 g/L
L-Valine 99%	8 g/L
**Bases**	
Adenine	0.5 g/L
Cytosine	0.5 g/L
Guanine 99%	0.5 g/L
Thymine	2g/L
Uracil	0.5 g/L
**Vitamins solution**	
Biotin	5 mg/L
D-Calcium pantothenate 98%	0.25 g/L
Nicotinic acid 99.5%	1.2 g/L
Thiamine hydrochloride	1 g/L
**Trace elements**	
Calcium chloride dihydrate	0.5 g/L
Cobalt chloride hexahydrate	0.4 g/L
Copper (II) sulfate pentahydrate	0.05 g/L
Iron (III) chloride hexahydrate	8 g/L
Manganese (II) sulfate monohydrate	0.56 g/L
Nickel (II) chloride hexahydrate	0.023 g/L
Zinc chloride ≥ 98%	0.0695 g/L
**Carbon source**	
Glucose anhydrous	4 g/L
**Ultrapure double-distilled deionized water to**	1L

**Table 4:** The sequence of real-time PCR primers for the selected virulence and regulatory genes, annealing temperatures, and expected amplicons sizes.

Gene Name	Gene Abbreviation	Primer Sequence 5′–3′ Forward and Reverse	Annealing Temperature °C	Expected Amplicon Size
Accessory gene regulator	*agrA*	CAAAGTTGCAGCGATGGATTT	62	92
		AGCGTGTATGTGCAGTTTCT		
Alpha-hemolysin	*hla*	GGCTCTATGAAAGCAGCAGAT	61	88
		CTGTAGCGAAGTCTGGTGAAA		
Beta-hemolysin	*hlb*	ATCCTTACCAAACACCTGTACTC	60	269
		AGCACCACAACGTGAATCT		
Delta-hemolysin	*hld*	GGAGTGATTTCAATGGCACAAG	60	83
		GTGAATTTGTTCACTGTGTCGATAA		
Clumping factor A	*clfA*	GAATCAGCTCCACAGAGTACAG	62	106
		CAGCTACTGCCGCTAAACTAA		
Fibronectin binding protein B	*fnbpB*	AGCTCAACCAAGTAACGTCTC	62	114
		ACATCTGTACCTGTCGCTTTAG		
Gyrase enzyme	*gyr A*	TCCCAACTGCTGGACTTATTT	62	111
		CGCCTCCACGTTCTTCAATA		
Intercellular Adhesion molecule C	*icaC*	GCGTTAGCAAATGGAGACTATTG	63	79
		GCGTGCAAATACCCAAGATAAC		
Intercellular Adhesion molecule D	*icaD*	AAGCCCAGACAGAGGGAATA	62	85
		AGACACAAGATATAGCTAAGTGC		
Murein hydrolase activator A	*cidA*	GTACCGCTAACTTGGGTAGAAG	62	109
		GCGTAATTTCGGAAGCAACAT		
Murein hydrolase activator B	*cidB*	ACGCAACGGTCGTATGTTTAG	63	105
		TCAGCATGACGCCAGTTAATAC		
Sigma factor B	*sigB*	AAGGACAATCACATCACGAAGA	62	101
		GGCTTCAAACTTCCGTTCAAA		
Zinc metalloprotease Aureolysin	*aur1*	GGTCGCACATTCACAAGTTTATC	62	84
		CGCCTGACTGGTCCTTATATTC		
16s Ribosomal RNA	*16s*	TGAGATGTTGGTTAAGTCCCGCA	60	188
		CGGTTTCGCTGCCCTTTGTATTGT		

## Data Availability

The data presented in this study are available in the main text, figures, tables and Supplementary Material.

## Supplementary Materials

The following are available online at https://www.mdpi.com/article/10.3390/pathogens10101228/s1. Table S1: Zinc concentrations in CDM using inductively coupled plasma spectrometry (ICP). Figure S1: Effect of different zinc concentrations on the growth of *S. aureus* standard MSSA RN6390, MRSA N315 strains, and MRSA clinical isolate at different time points. Figure S2: Assessment of catalase activity of *S. aureus*. Figure S3: Assessment of protease activity of *S. aureus*. Figure S4: Calibration curve of zinc concentration using Zincon assay.

## References

[1] Holden M., Lindsay J., Bentley S. (2006). The Grapes of Wrath. Nat. Rev. Microbiol..

[2] Schito G.C. (2006). The Importance of the Development of Antibiotic Resistance in *Staphylococcus aureus*. Clin. Microbiol. Infect..

[3] Costa A.R., Batistão D.W.F., Ribas R.M., Sousa A.M., Pereira O., Botelho C.M. (2013). *Staphylococcus aureus* Virulence Factors and Disease. Microbial Pathogens and Strategies for Combating Them: Science, Technology and Education.

[4] Udo E.E. (2013). Community-Acquired Methicillin-Resistant *Staphylococcus aureus*: The New Face of an Old Foe?. Med. Princ. Pract..

[5] Abouelfetouh A. (2017). The Status of Methicillin Resistance Among Egyptian *Staphylococcus aureus* Isolates: An Overview. Infect. Disord.-Drug Targets.

[6] Kong E.F., Johnson J.K., Jabra-Rizk M.A. (2016). Community-Associated Methicillin-Resistant *Staphylococcus aureus*: An Enemy amidst Us. PLoS Pathog..

[7] Lee A.S., De Lencastre H., Garau J., Kluytmans J., Malhotra-Kumar S., Peschel A., Harbarth S. (2018). Methicillin-Resistant *Staphylococcus aureus*. Nat. Rev. Dis. Primers.

[8] Dinges M.M., Orwin P.M., Schlievert P.M. (2000). Exotoxins of *Staphylococcus aureus*. Clin. Microbiol. Rev..

[9] DeLeo F.R., Diep B.A., Otto M. (2009). Host Defense and Pathogenesis in *Staphylococcus aureus* Infections. Infect. Dis. Clin. N. Am..

[10] Fey P.D., Olson M.E. (2010). Current Concepts in Biofilm Formation of *Staphylococcus epidermidis*. Future Microbiol..

[11] Otto M. (2008). Staphylococcal Biofilms. Curr. Top. Microbiol. Immunol..

[12] Soto S.M. (2014). Importance of Biofilms in Urinary Tract Infections: New Therapeutic Approaches. Adv. Biol..

[13] Trautner B., Darouiche R. (2004). Role of Biofilm in Catheter-Associated Urinary Tract Infection. Am. J. Infect. Control.

[14] Weinberg E.D. (1974). Iron and Susceptibility to Infectious Disease. Science.

[15] Corbin B.D., Seeley E.H., Raab A., Feldmann J., Miller M.R., Torres V.J., Anderson K.L., Dattilo B.M., Dunman P.M., Gerads R. (2008). Metal Chelation and Inhibition of Bacterial Growth in Tissue Abscesses. Science.

[16] Agranoff D.D., Krishna S. (1998). Metal Ion Homeostasis and Intracellular Parasitism. Mol. Microbiol..

[17] Hood M.I., Skaar E.P. (2012). Nutritional Immunity: Transition Metals at the Pathogen–Host Interface. Nat. Rev. Microbiol..

[18] Mcdevitt C.A., Ogunniyi A.D., Valkov E., Lawrence M.C., Kobe B., Mcewan A.G., Paton J.C. (2011). A Molecular Mechanism for Bacterial Susceptibility to Zinc. PLoS Pathog..

[19] Hammer N.D., Skaar E.P. (2012). The Impact of Metal Sequestration on *Staphylococcus aureus* Metabolism. Curr. Opin. Microbiol..

[20] Festa R.A., Thiele D.J. (2012). Copper at the Front Line of the Host-Pathogen Battle. PLoS Pathog..

[21] Cassat J.E., Skaar E.P. (2012). Metal Ion Acquisition in *Staphylococcus aureus*: Overcoming Nutritional Immunity. Semin. Immunopathol..

[22] Beasley F.C., Vinés E.D., Grigg J.C., Zheng Q., Liu S., Lajoie G.A., Murphy M.E.P., Heinrichs D.E. (2009). Characterization of Staphyloferrin A Biosynthetic and Transport Mutants in *Staphylococcus aureus*. Mol. Microbiol..

[23] Skaar E.P. (2010). The Battle for Iron between Bacterial Pathogens and Their Vertebrate Hosts. PLoS Pathog..

[24] Ciccaglione A.F., Di Giulio M., Di Lodovico S., Di Campli E., Cellini L., Marzio L. (2019). Bovine Lactoferrin Enhances the Efficacy of Levofloxacin-Based Triple Therapy as First-Line Treatment of *Helicobacter pylori* Infection: An in Vitro and in Vivo Study. J. Antimicrob. Chemother..

[25] Kehl-Fie T.E., Chitayat S., Hood M.I., Damo S., Restrepo N., Garcia C., Munro K.A., Chazin W.J., Skaar E.P. (2011). Nutrient Metal Sequestration by Calprotectin Inhibits Bacterial Superoxide Defense, Enhancing Neutrophil Killing of *Staphylococcus aureus*. Cell Host Microbe.

[26] Rahman M.T., Karim M.M. (2018). Metallothionein: A Potential Link in the Regulation of Zinc in Nutritional Immunity. Biol. Trace Elem. Res..

[27] Chowdhury D., Alrefai H., Landero Figueroa J.A., Candor K., Porollo A., Fecher R., Divanovic S., Deepe G.S., Subramanian Vignesh K. (2019). Metallothionein 3 Controls the Phenotype and Metabolic Programming of Alternatively Activated Macrophages. Cell Rep..

[28] Gaddy J.A., Radin J.N., Loh J.T., Piazuelo M.B., Kehl-Fie T.E., Delgado A.G., Ilca F.T., Peek R.M., Cover T.L., Chazin W.J. (2014). The Host Protein Calprotectin Modulates the *Helicobacter pylori* Cag Type IV Secretion System via Zinc Sequestration. PLoS Pathog..

[29] Velasco E., Wang S., Sanet M., Fernández-Vázquez J., Jové D., Glaría E., Valledor A.F., O’Halloran T.V., Balsalobre C. (2018). A New Role for Zinc Limitation in Bacterial Pathogenicity: Modulation of α-Hemolysin from Uropathogenic *Escherichia coli*. Sci. Rep..

[30] Hussain M., Hastings J.G.M., White P.J. (1991). A Chemically Defined Medium for Slime Production by Coagulase-Negative Staphylococci. J. Med. Microbiol..

[31] Lindsay J.A., Foster S.J. (2001). Zur: A Zn2+-Responsive Regulatory Element of *Staphylococcus aureus* The GenBank Accession Number for the Sequence Reported in This Paper Is AF101263. Microbiology.

[32] Conrady D.G., Brescia C.C., Horii K., Weiss A.A., Hassett D.J., Herr A.B. (2008). A Zinc-Dependent Adhesion Module Is Responsible for Intercellular Adhesion in Staphylococcal Biofilms. Proc. Natl. Acad. Sci. USA.

[33] Grim K.P., San Francisco B., Radin J.N., Brazel E.B., Kelliher J.L., Párraga Solórzano P.K., Kim P.C., McDevitt C.A., Kehl-Fie T.E. (2017). The Metallophore Staphylopine Enables *Staphylococcus aureus* To Compete with the Host for Zinc and Overcome Nutritional Immunity. mBio.

[34] Li K., Gifford A.H., Hampton T.H., O’Toole G.A. (2020). Availability of Zinc Impacts Interactions between Streptococcus Sanguinis and Pseudomonas Aeruginosa in Coculture. J. Bacteriol..

[35] Taylor A. (1997). Measurement of Zinc in Clinical Samples. Ann. Clin. Biochem..

[36] Hussain W., Mumtaz A., Yasmeen F., Khan S.Q., Butt T. (2014). Reference Range of Zinc in Adult Population (20–29 years) of Lahore, Pakistan. Pak. J. Med Sci..

[37] Kaur K., Gupta R., Saraf S.A., Saraf S.K. (2014). Zinc: The Metal of Life. Compr. Rev. Food Sci. Food Saf..

[38] Kambe T., Hashimoto A., Fujimoto S. (2014). Current Understanding of ZIP and ZnT Zinc Transporters in Human Health and Diseases. Cell. Mol. Life Sci..

[39] Plum L.M., Rink L., Hajo H. (2010). The Essential Toxin: Impact of Zinc on Human Health. Int. J. Environ. Res. Public Health.

[40] Bose J.L., Daly S.M., Hall P.R., Bayles K.W. (2014). Identification of the *Staphylococcus aureus* VfrAB Operon, a Novel Virulence Factor Regulatory Locus. Infect. Immun..

[41] Lin Y.-C., Peterson M.L. (2010). New Insights into the Prevention of Staphylococcal Infections and Toxic Shock Syndrome. Expert Rev. Clin. Pharmacol..

[42] Palmer L.D., Skaar E.P. (2016). Transition Metals and Virulence in Bacteria. Annu. Rev. Genet..

[43] Weiss G., Carver P.L. (2018). Role of Divalent Metals in Infectious Disease Susceptibility and Outcome. Clin. Microbiol. Infect..

[44] Platte J.A., Marcy V.M. (1959). Photometric Determination of Zinc with Zincon. Application to Water Containing Heavy Metals. Anal. Chem..

[45] Olesik J.W. (2020). ICP-OES Capabilities, Developments, Limitations, and Any Potential Challengers?. Spectroscopy.

[46] Soltyk K., Lozak A., Warowna-Grzeskiewicz M., Fijalek Z. (2000). The AAS ICP-MS and Electrochemical Determinations of Zinc in Selected Pharmaceutical Preparations. Acta Pol. Pharm..

[47] Zecconi A., Scali F. (2013). *Staphylococcus aureus* Virulence Factors in Evasion from Innate Immune Defenses in Human and Animal Diseases. Immunol. Lett..

[48] Atshan S.S., Shamsudin M.N., Karunanidhi A., van Belkum A., Lung L.T.T., Sekawi Z., Nathan J.J., Ling K.H., Seng J.S.C., Ali A.M. (2013). Quantitative PCR Analysis of Genes Expressed during Biofilm Development of Methicillin Resistant *Staphylococcus aureus* (MRSA). Infect. Genet. Evol..

[49] Conrady D.G., Wilson J.J., Herr A.B. (2013). Structural Basis for Zn2+-Dependent Intercellular Adhesion in Staphylococcal Biofilms. Proc. Natl. Acad. Sci. USA.

[50] Formosa-Dague C., Speziale P., Foster T.J., Geoghegan J.A., Dufrêne Y.F. (2016). Zinc-Dependent Mechanical Properties of *Staphylococcus aureus* Biofilm-Forming Surface Protein SasG. Proc. Natl. Acad. Sci. USA.

[51] Rogers S.A., Huigens R.W., Melander C. (2009). A 2-Aminobenzimidazole That Inhibits and Disperses Gram-Positive Biofilms through a Zinc-Dependent Mechanism. J. Am. Chem. Soc..

[52] Machado H., Weng L.L., Dillon N., Seif Y., Holland M., Pekar J.E., Monk J.M., Nizet V., Palsson B.O., Feist A.M. (2019). Strain-Specific Metabolic Requirements Revealed by a Defined Minimal Medium for Systems Analyses of *Staphylococcus aureus*. Appl. Environ. Microbiol..

[53] Tam K., Torres V.J. (2019). *Staphylococcus aureus* Secreted Toxins and Extracellular Enzymes. Gram-Positive Pathogens.

[54] Mukherji R., Patil A., Prabhune A. (2015). Role of Extracellular Proteases in Biofilm Disruption of Gram Positive Bacteria with Special Emphasis on *Staphylococcus aureus* Biofilms. Enzym. Eng..

[55] Beaufort N., Wojciechowski P., Sommerhoff C.P., Szmyd G., Dubin G., Eick S., Kellermann J., Schmitt M., Potempa J., Magdolen V. (2008). The Human Fibrinolytic System Is a Target for the Staphylococcal Metalloprotease Aureolysin. Biochem. J..

[56] Laarman A.J., Ruyken M., Malone C.L., van Strijp J.A.G., Horswill A.R., Rooijakkers S.H.M. (2011). *Staphylococcus aureus* Metalloprotease Aureolysin Cleaves Complement C3 To Mediate Immune Evasion. J. Immunol..

[57] Takeuchi S., Saito M., Imaizumi K., Kaidoh T., Higuchi H., Inubushi S. (2002). Genetic and Enzymatic Analyses of Metalloprotease (Aureolysin) from Staphylococcus aureus Isolated from Domestic Animals. Vet. Microbiol..

[58] Pané-Farré J., Jonas B., Förstner K., Engelmann S., Hecker M. (2006). The SigB Regulon in *Staphylococcus aureus* and Its Regulation. Int. J. Med Microbiol..

[59] Vandenesch F., Lina G., Henry T. (2012). *Staphylococcus aureus* Hemolysins, Bi-Component Leukocidins, and Cytolytic Peptides: A Redundant Arsenal of Membrane-Damaging Virulence Factors?. Front. Cell. Infect. Microbiol..

[60] Berube B.J., Wardenburg J.B. (2013). *Staphylococcus aureus* α-Toxin: Nearly a Century of Intrigue. Toxins.

[61] Wilke G.A., Wardenburg J.B. (2010). Role of a Disintegrin and Metalloprotease 10 in *Staphylococcus aureus* α-Hemolysin-Mediated Cellular Injury. Proc. Natl. Acad. Sci. USA.

[62] Menestrina G., Bashford C.L., Pasternak C.A. (1990). Pore-Forming Toxins: Experiments with *S. aureus* α-Toxin, *C. perfringens* θ-Toxin and *E. coli* Haemolysin in Lipid Bilayers, Liposomes and Intact Cells. Toxicon.

[63] Titball R.W. (1993). Bacterial Phospholipases, C. Microbiol. Rev..

[64] Laffan A.M., McKenzie R., Forti J., Conklin D., Marcinko R., Shrestha R., Bellantoni M., Greenough W.B. (2011). Lactoferrin for the Prevention of Post-Antibiotic Diarrhoea. J. Health Popul. Nutr..

[65] Chilton C.H., Crowther G.S., Śpiewak K., Brindell M., Singh G., Wilcox M.H., Monaghan T.M. (2016). Potential of Lactoferrin to Prevent Antibiotic-Induced *Clostridium difficile* Infection. J. Antimicrob. Chemother..

[66] MacManus C.F., Collins C.B., Nguyen T.T., Alfano R.W., Jedlicka P., de Zoeten E.F. (2017). VEN-120, a Recombinant Human Lactoferrin, Promotes a Regulatory T Cell [Treg] Phenotype and Drives Resolution of Inflammation in Distinct Murine Models of Inflammatory Bowel Disease. J. Crohn’s Colitis.

[67] Pall E., Roman A. (2020). Lactoferrin Functionalized Biomaterials: Tools for Prevention of Implant-Associated Infections. Antibiotics.

[68] Wilson D., Varigos G., Ackland M.L. (2006). Apoptosis May Underlie the Pathology of Zinc-Deficient Skin. Immunol. Cell Biol..

[69] Novick R.P. (1991). Genetic Systems in Staphylococci. Methods Enzymol..

[70] Kuroda M., Ohta T., Uchiyama I., Baba T., Yuzawa H., Kobayashi I., Cui L., Oguchi A., Aoki K., Nagai Y. (2001). Whole Genome Sequencing of Meticillin-Resistant *Staphylococcus aureus*. Lancet.

[71] Nour El-Din H.T., Yassin A.S., Ragab Y.M., Hashem A.M. (2021). Phenotype-Genotype Characterization and Antibiotic-Resistance Correlations Among Colonizing and Infectious Methicillin-Resistant *Staphylococcus aureus* Recovered from Intensive Care Units. Infect. Drug Resist..

[72] CLSI (2014). Performance Standards for Antimicrobial Susceptibility Testing.

[73] Vitko N.P., Richardson A.R. (2013). Laboratory Maintenance of Methicillin-Resistant *Staphylococcus aureus* (MRSA). Curr. Protoc. Microbiol..

[74] Taylor D., Holland K. (1989). Amino Acid Requirements for the Growth and Production of Some Exocellular Products of *Staphylococcus aureus*. J. Appl. Bacteriol..

[75] Remy L., Carrière M., Derré-bobillot A., Martini C., Sanguinetti M., Borezée-durant E., Yvette G. (2013). The *Staphylococcus aureus* Opp1 ABC Transporter Imports Nickel and Cobalt in Zinc-Depleted Conditions and Contributes to Virulence. Mol. Microbiol..

[76] Naves P., del Prado G., Huelves L., Gracia M., Ruiz V., Blanco J., Rodrguez-Cerrato V., Ponte M.C., Soriano F. (2008). Measurement of Biofilm Formation by Clinical Isolates of *Escherichia coli* Is Method-Dependent. J. Appl. Microbiol..

[77] Reiner K. (2010). Catalase-Test-Protocol. Am. Soc. Microbiol..

[78] Kong C., Chee C.-F., Richter K., Thomas N., Rahman N.A., Nathan S. (2018). Suppression of *Staphylococcus aureus* Biofilm Formation and Virulence by a Benzimidazole Derivative, UM-C162. Sci. Rep..

[79] Kateete D.P., Kimani C.N., Katabazi F.A., Okeng A., Okee M.S., Nanteza A., Joloba M.L., Najjuka F.C. (2010). Identification of *Staphylococcus aureus*: DNase and Mannitol Salt Agar Improve the Efficiency of the Tube Coagulase Test. Ann. Clin. Microbiol. Antimicrob..

[80] Bernheimer A.W. (1988). [30] Assay of Hemolytic Toxins. Microbial Toxins: Tools in Enzymology.

[81] Costabile M. (2010). Measuring the 50% Haemolytic Complement (CH_50_) Activity of Serum. J. Vis. Exp..

[82] Attia A.S., Benson M.A., Stauff D.L., Torres V.J., Skaar E.P. (2010). Membrane Damage Elicits an Immunomodulatory Program in *Staphylococcus aureus*. PLoS Pathog..

[83] Kong C., Neoh H.M., Nathan S. (2016). Targeting *Staphylococcus aureus* Toxins: A Potential Form of Anti-Virulence Therapy. Toxins.

[84] Zhou J.L. (1999). Zn Biosorption by *Rhizopus arrhizus* and Other Fungi. Appl. Microbiol. Biotechnol..

