# Supplementary material for: Zinc Deprivation as a Promising Approach for Combating Methicillin-Resistant Staphylococcus aureus: A Pilot Study
Source: Pathogens. 2021 Sep 23;10(10):1228. doi: 10.3390/pathogens10101228 (PMC8540720; doi:10.3390/pathogens10101228)
Supplement: Supplementary file 1 [file pathogens-10-01228-s001.zip › pathogens-1327939-SI.pdf]

Table S1. Zinc concentrations in CDM using inductively coupled plasma (ICP) spectrometry

**Intended $ZnCl_2$ concentration in the prepared CDM in μM and its equivalent calculated concentration in ppm***	**Actual $ZnCl_2$ concentration in the prepared CDM as measured by ICP in ppm and its equivalent calculated concentration in μM**
**0** μM ≡ 0 ppm	0.007 ppm ≡ 0.05 μM
7 μM ≡ 0.95 ppm	1.50 ppm ≡ 11 μM
20 μM ≡ 2.73 ppm	2.70 ppm ≡ 19.8 μM (~20 μM)
50 μM ≡ 6.81 ppm	6.10 ppm ≡ 45 μM
500 μM ≡ 68.14 ppm	69.60 ppm ≡ 510 μM

*Intended and actual $ZnCl_2$ concentrations were statistically insignificant using Student t-test.

(a)

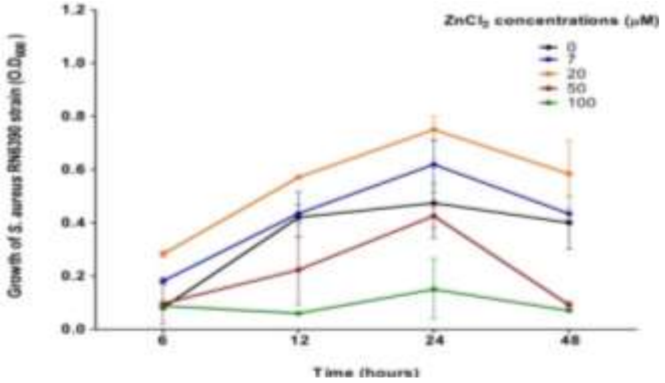

(b)

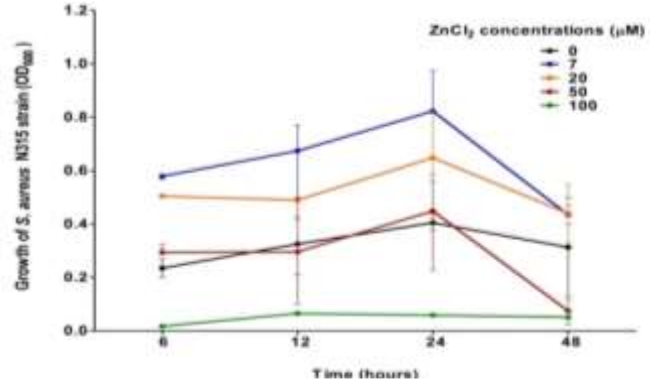

(c)

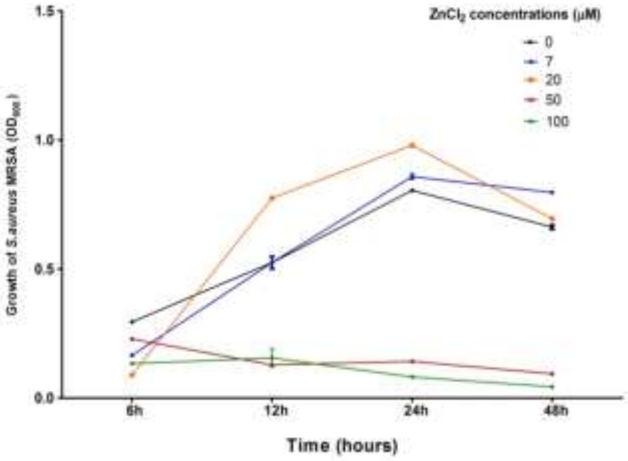

Figure S1. Effect of different zinc concentrations on the growth of *S. aureus* standard MSSA strain RN6390, standard MRSA strain N315 and MRSA clinical isolate at different time points. Growth pattern of *S. aureus* over 48h showed maximum effect of $ZnCl_2$ limitation at 24h compared to physiological concentration range of Zn (7–20 μM). (a) RN6390 standard MSSA strain, (b) N315 standard MRSA strain and (c) MRSA clinical isolate. Mann-Whitney test was used for statistical significance of the medians at $p < 0.05$. Data represented as medians with interquartile range of at least 4 separate experiments.

(a)

(b)

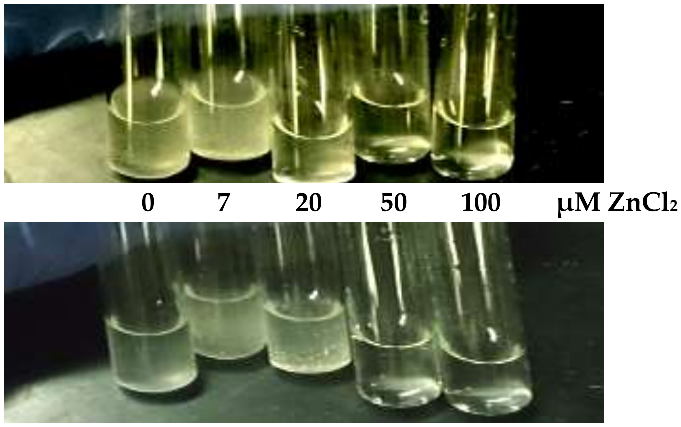

Figure S2. Assessment of catalase activity of *S. aureus* (a) RN6390 strain and (b) MRSA clinical isolate. Catalase activity assessed in CDM, tubes were arranged according to zinc concentrations in CDM as follows; 0, 7, 20, 50, and 100 µM. Better effervescence was observed at physiological range of zinc (7-20 µM), while zinc limitation and high concentrations showed little or no effervescence, respectively.

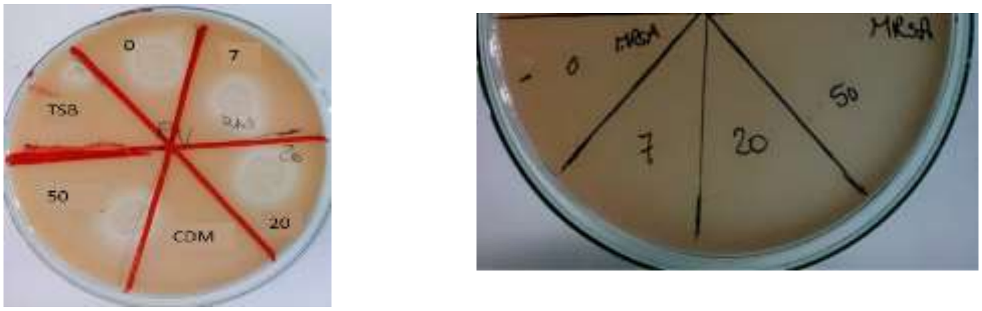

(a) (b)

Figure S3. Assessment of protease activity on skimmed milk agar of *S. aureus* (a) RN6390 strain and (b) MRSA clinical isolate. Cultures of RN6390 showed equal zones of protease activity on skimmed milk, while MRSA showed no protease activity. Cultures were grown in CDM with different zinc concentrations (0, 7, 20, and 50 µM) as indicated on the plate showed no differences in protease activity on skimmed milk agar based on zinc levels.

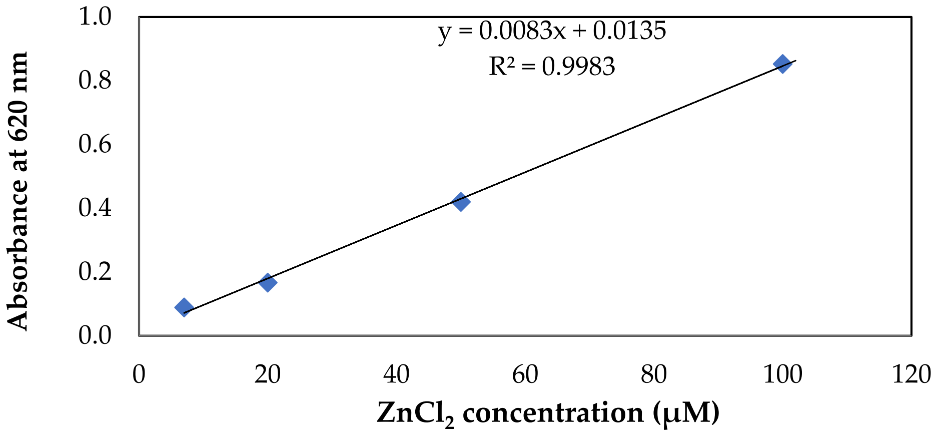

Figure S4. Calibration curve of zinc concentration using Zincon assay. Prepared samples containing the following known amounts of zinc: 7, 20, 50, 100 µM $ZnCl_2$ were treated as stated under the Zincon assay and the absorbance was measured at 620 nm. The CDM without zinc gave absorbance of 0.015 at 620 nm, according to the standard curve "$y = 0.0083x + 0.0135$" corresponded to 0.0245 ppm $ZnCl_2 \equiv 0.18$ µM.
